# Supplementary material for: Body Adiposity Partially Mediates the Association between FTO rs9939609 and Lower Adiponectin Levels in Chilean Children
Source: Children (Basel). 2023 Feb 22;10(3):426. doi: 10.3390/children10030426 (PMC10047575; doi:10.3390/children10030426)
Supplement: Supplementary file 1 [file children-10-00426-s001.zip › children-2199819-supplementary.pdf]

Body adiposity partially mediates the association between FTO rs9939609 and lower adiponectin levels in Chilean children

Carolina Ochoa-Rosales, Lorena Mardones, Marcelo Villagrán, Claudio Aguayo, Miquel Martorell, Benilde Riffo, Carlos Celis-Morales and Natalia Ulloa, on behalf of ELHOC Consortium

SUPPLEMENTARY FIGURES

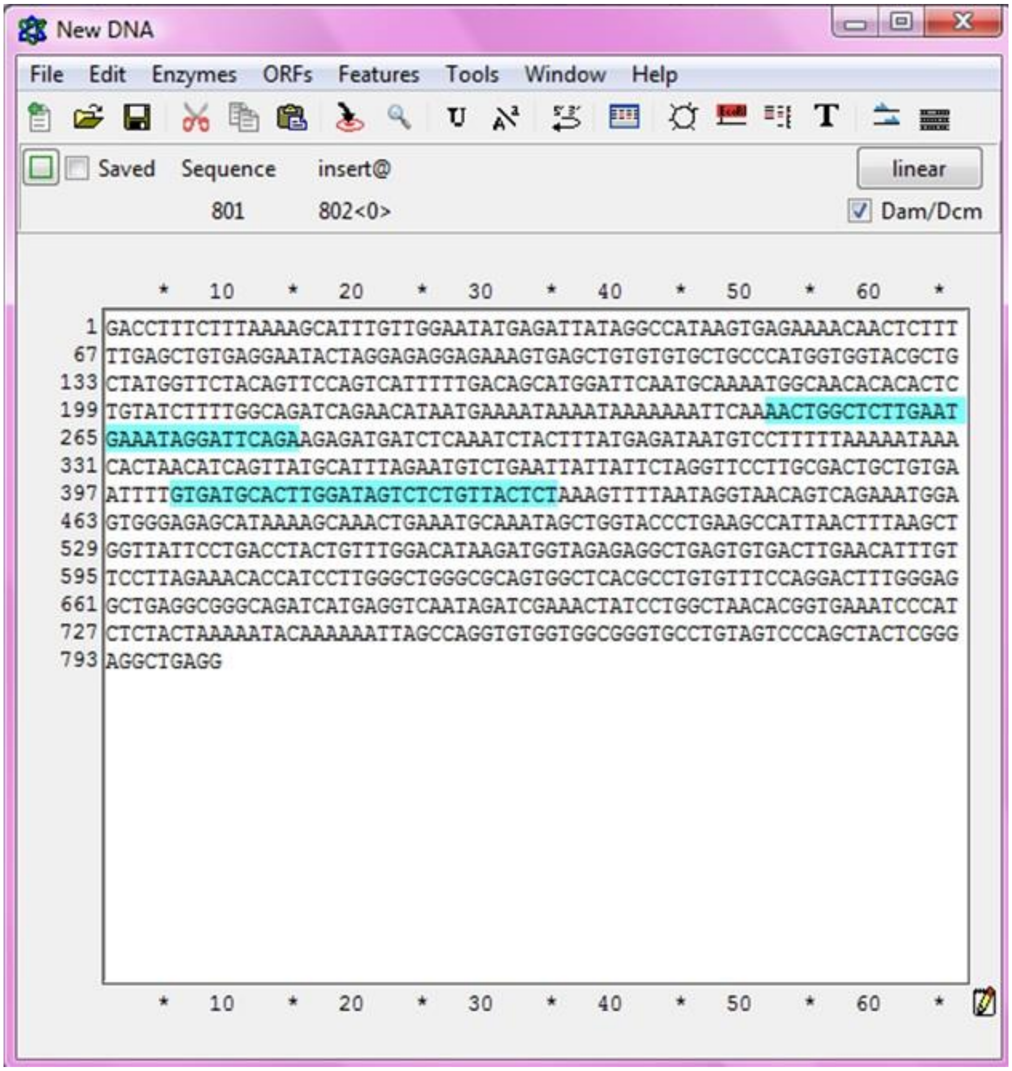

Supplementary Figure S-1: The figure shows the sequence of the polymerase chain reaction (PCR) product. Sequencies highlighted in calypso color represent forward (5′ AACTGGCTCTTGAATGAAATAGGATTCAGA 3′) and reverse primers (5′-GTGATGCACTTGGATAGTCTCTGTTACTCT-3′).

a)

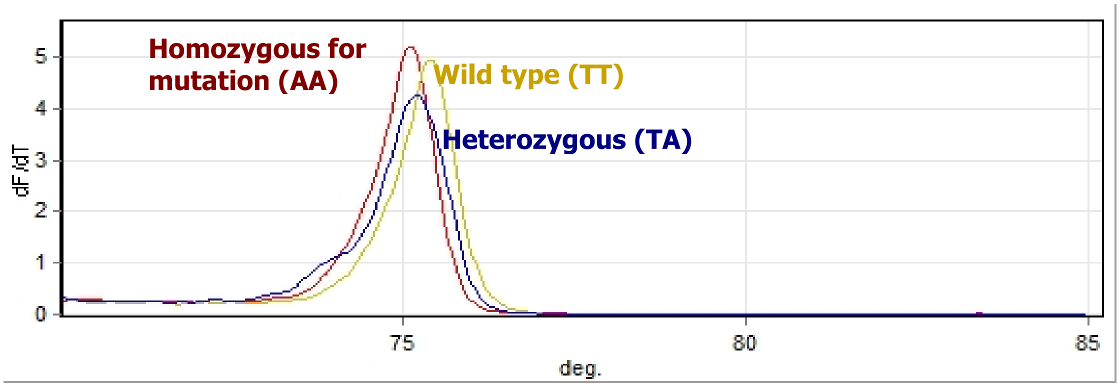

b)

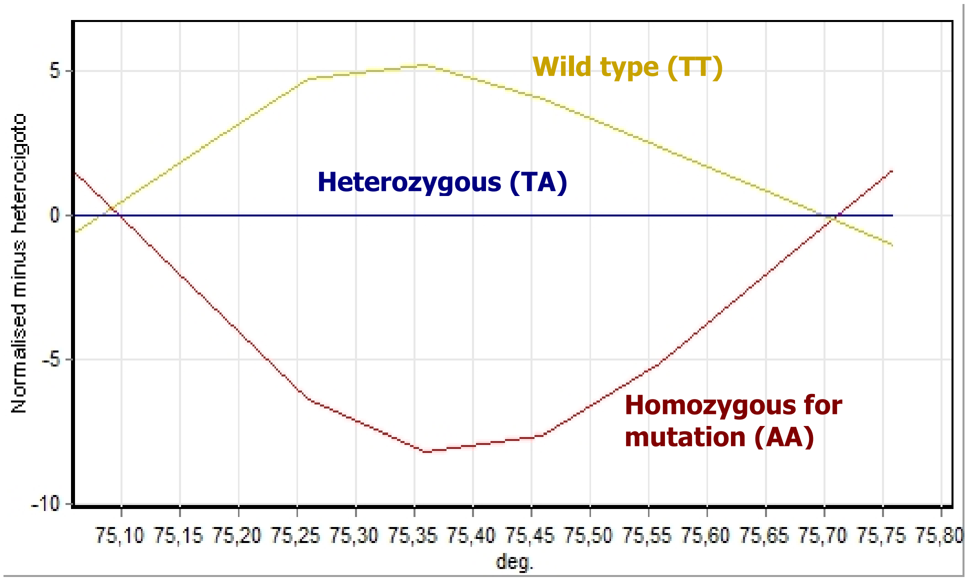

Supplementary Figure S-2: High Resolution Melting (HRM) curves for genetic variants of *FTO* rs9939609. Figure S-2 a) shows the differential melting curves, one for each genotype. Figure S-2 b) shows the differential melting curves normalized with respect to the heterozygous genotype (TA).

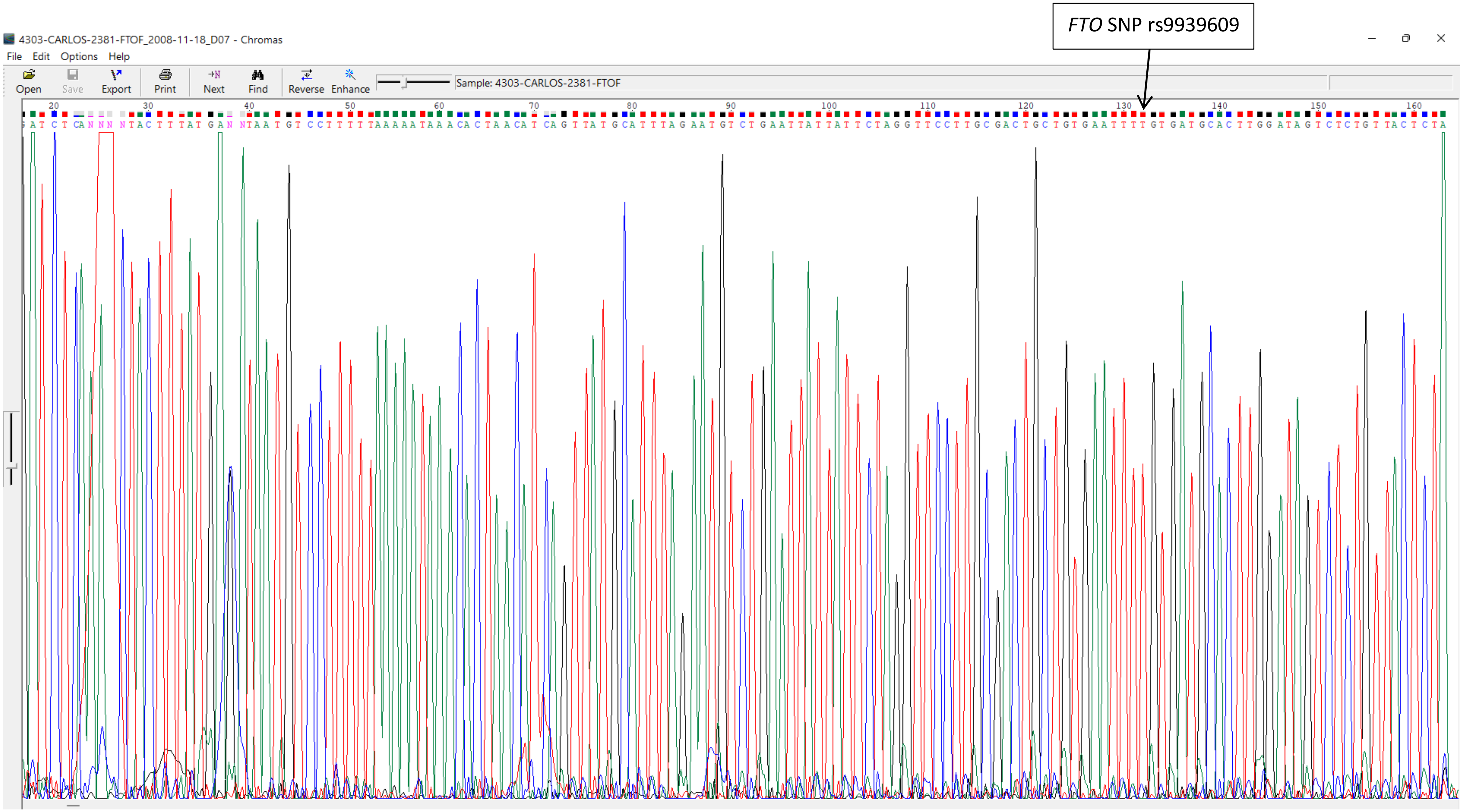

Supplementary Figure S-3: Figure shows the sequencing data of one of the control samples owning a wild type allele (T)
